# Hydrogels for the management of second-degree burns: currently available options and future promise

**DOI:** 10.1093/burnst/tkac047

**Published:** 2022-12-08

**Authors:** Katherine A Cook, Edith Martinez-Lozano, Robert Sheridan, Edward K Rodriguez, Ara Nazarian, Mark W Grinstaff

**Affiliations:** Department of Chemistry, Biomedical Engineering, and Medicine, Boston University, Boston, MA, 02215, USA; Musculoskeletal Translational Innovation Initiative, Carl J. Shapiro, Department of Orthopaedic Surgery, Beth Israel Deaconess Medical Center and Harvard Medical School, Boston, MA, 02215, USA; Shriners Hospitals for Children and Burns Service, Department of Surgery, Massachusetts General Hospital, Boston, MA, 02214, USA; Musculoskeletal Translational Innovation Initiative, Carl J. Shapiro, Department of Orthopaedic Surgery, Beth Israel Deaconess Medical Center and Harvard Medical School, Boston, MA, 02215, USA; Musculoskeletal Translational Innovation Initiative, Carl J. Shapiro, Department of Orthopaedic Surgery, Beth Israel Deaconess Medical Center and Harvard Medical School, Boston, MA, 02215, USA; Department of Orthopaedic Surgery, Yerevan State Medical University, Yerevan, Armenia; Department of Chemistry, Biomedical Engineering, and Medicine, Boston University, Boston, MA, 02215, USA

**Keywords:** Burn, Wound, Hydrogel, Dressing, Second-degree

## Abstract

Burn wounds result from exposure to hot liquids, chemicals, fire, electric discharge or radiation. Wound severity ranges from first-degree injury, which is superficial, to fourth-degree injury, which exposes bone, tendons and muscles. Rapid assessment of burn depth and accurate wound management in the outpatient setting is critical to prevent injury progression into deeper layers of the dermis. Injury progression is of particular pertinence to second-degree burns, which are the most common form of thermal burn. As our understanding of wound healing advances, treatment options and technologies for second-degree burn management also evolve. Polymeric hydrogels are a class of burn wound dressings that adhere to tissue, absorb wound exudate, protect from the environment, can be transparent facilitating serial wound evaluation and, in some cases, enable facile removal for dressing changes. This review briefly describes the burn level classification and common, commercially available dressings used to treat second-degree burns, and then focuses on new polymeric hydrogel burn dressings under preclinical development analyzing their design, structure and performance. The review presents the follow key learning points: (1) introduction to the integument system and the wound-healing process; (2) classification of burns according to severity and clinical appearance; (3) available dressings currently used for second-degree burns; (4) introduction to hydrogels and their preparation and characterization techniques; and (5) pre-clinical hydrogel burn wound dressings currently being developed.

## Background

### Anatomy of the skin and wound healing

The integument, or skin, the largest organ in the human body, is a physical barrier to injuries and environmental pathogens that maintains homeostasis, modulates inflammation and transmits tactile sensations [1]. Burn injuries can disrupt any of the skin’s three anatomic layers: the epidermis, dermis and/or hypodermis. Epidermal cells, composing the outer surface of the skin, regenerate from cells deep within the dermal appendages (including hair follicles, sebaceous glands and sweat glands) and from adipose-derived stem cells found on the hypodermis [1–5]. The tightly regulated contiguous healing process involves four phases: (1) hemostasis, (2) inflammation, (3) cellular proliferation and (4) matrix remodeling, and ultimately results in a cross-linking of collagen I fibers to confer adequate tensile strength to the newly formed scar [6,7]. An in-depth background of the integument and the wound healing process has been described previously by Lloyd *et al.*, and the reader is referred to this review [7].

### Classification

**Figure 1.**
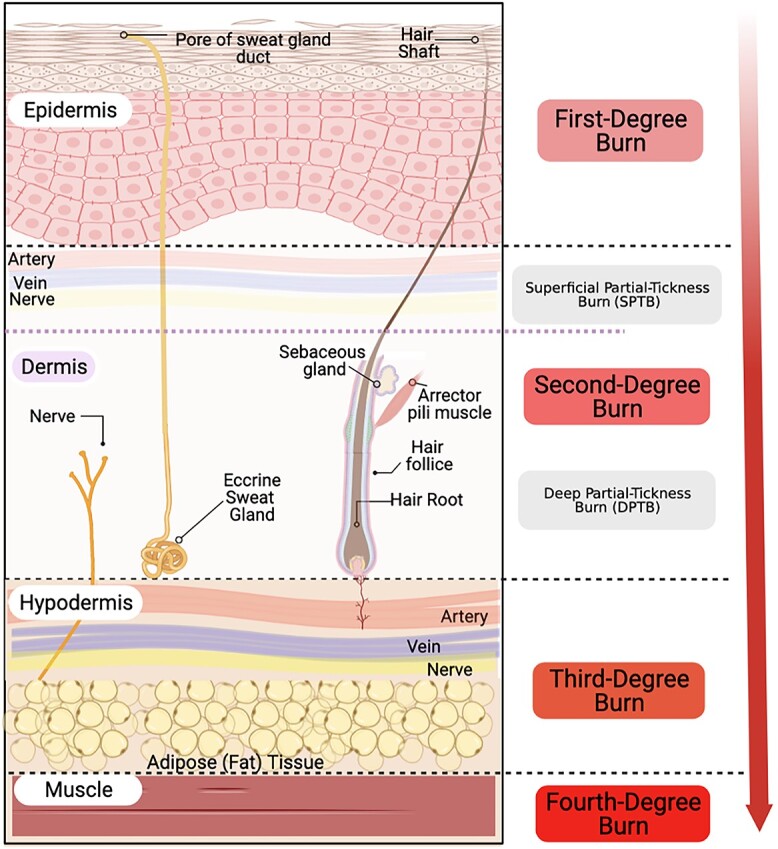
Anatomy and classification of burn wound depth. Created with Biorender.com

**Figure 2.**
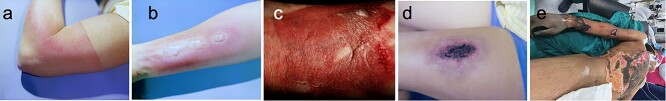
Clinical examples of burn degrees and their associated nomenclature. (**a**) First-degree burn commonly referred as ‘sunburn’. (**b**) Second-degree superficial partial-thickness burn. (**c**) Second-degree deep partial-thickness burn. (**d**) Third-degree burn with eschar formation. (**e**) Fourth-degree burn affecting the rectus abdominus muscles. Images (a), (b) and (d) were obtained from the Public Health Image Library. Image (c) was purchased from Shutterstock.com while image (e) was Copyright permission obtained from Saied *et al.* [13]

**Figure 3.**
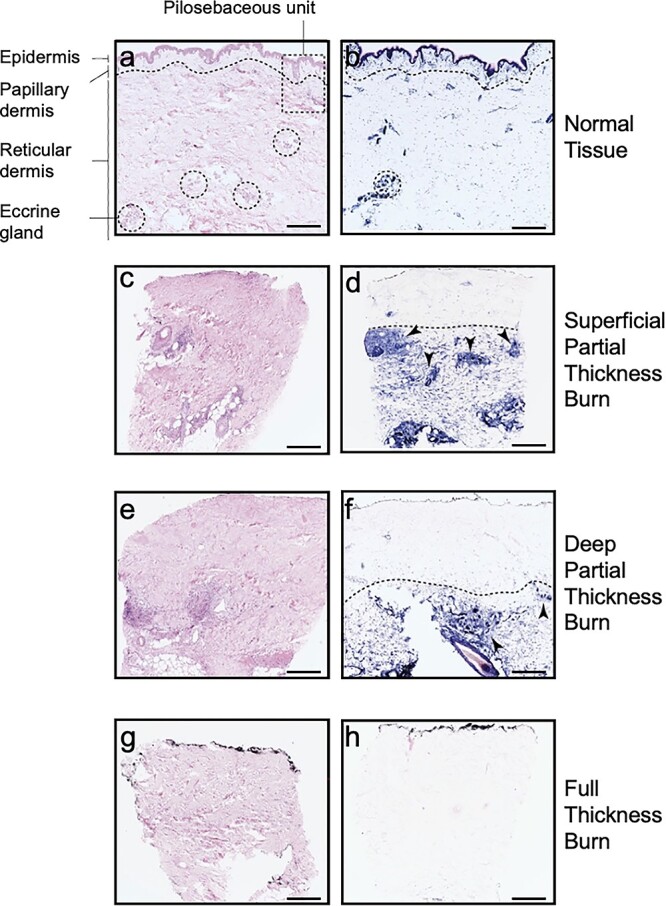
Histologic biopsies illustrating normal and burned human skin. Images (**a**), (**c**), (**e**) and (**g**) are hematoxylin and eosin (H&E) stained (scale bar 500 μm). Images (**b**), (**d**), (**f**) and (**h**) are lactate dehydrogenase (LDH) stained (scale bar 500 μm) with viable cells stained blue. Images (a) and (b) illustrate normal tissue histology, with a dotted line representing the interface between the papillary and reticular dermis. The pilosebaceous unit (PSU) (dotted rectangle), composed of a hair follicle, arrector pili muscle and associated sebaceous gland, extends from the epidermis into the dermis. Dotted circles represent eccrine gland structures, which together with the PSU form the regenerative niches needed for wound healing. Images (c) and (d) represent superficial partial-thickness burns (SPTB) with complete absence of epithelial cells and minor involvement of the papillary dermis. In contrast, images (e) and (f) depict a deep partial-thickness burn (DPTB) with severe loss of epidermal and dermal epithelium. Note that the arrows in both SPTB and DPTB point to the remaining regenerative characteristic of second-degree burns. Images (g) and (h) represent a full-thickness burn with no visible regenerative potential. Copyright permission obtained from Karim *et al*. [11]

Burns are classified according to burn depth in four degrees (Figure 1 and Table 1) [7–11]. First-degree burns (Figure 2a), such as sunburns, are superficial, red and painful injuries that only affect the epidermis and typically heal completely without the need for intervention. Second-degree burns penetrate the dermis and are therefore referred to as partial-thickness burns. These burns are further categorized into superficial partial-thickness burns (SPTBs) (Figure 2b and Figure 3c, d), which entirely injure the epidermis and part of the dermis, and deep partial-thickness burns (DPTBs) (Figure 2c and Figure 3e, f), which extend deeper into the dermis layer [8]. Wounds resulting from second-degree burns can be very sensitive and painful when touched due to exposure of intact, sensory nerve endings. Re-epithelialization depends on the level of degradation of the dermis and the number of damaged skin appendages. Therefore, first- and second-degree superficial burns heal by a process called primary intention (the epithelium restoration of continuity occurs directly with minimal granulation tissue) while second-degree and more severe burns heal by secondary intention (loss of skin appendages needed for reepithelization) often resulting in scarring and contractures [4,12]. Third-degree burns destroy all skin layers, including the underlying subcutaneous fat, and are therefore considered full-thickness burns. These burn wounds present no sensitivity to touch due to destruction of the dermal plexus nerves (Figure 2d and Figure 3g, h) [7]. Lastly, fourth-degree burns extend through all skin layers as well as to muscle, tendons and bones (Figure 2e), consequently affecting nerve endings [13]. These burns are the most challenging to treat and often require surgical debridement and grafting [14].

**Table 1:** Classification of burn wound’s depth. Information from [4, 7, 9–11]

	**First-degree**	**Second-degree**		**Third-degree**	**Fourth-degree**
Depth of burn	Epidermis (superficial)	SPTB Epidermis and upper 1/3 dermis	DPTB Epidermis and dermis, affecting appendages	Full thickness (including subcutaneous fat)	Extends into muscle, tendons and/or bone
Histologic findings	Loss of epidermal attachment to dermis Epidermal cells with nuclear pyknosis (shrinkage)	Loss of epidermal attachment to dermis Vacuolar cytoplasmic disintegration of the basal cell layer	Coagulation of epidermis and dermis Denatured collagen appears swollen and basophilic Exudative infiltrate may be seen Loss of regenerative niches	Carbonized surface with intense basophilic coagulated dermis. Scant epidermal/dermal cells present Loss of regenerative niches	
Most common causes	Sunburn (prolonged UV exposure)	Brief contact with hot liquids, chemicals, flames or electric discharge (such as lightning)		Exposure to hot liquids, chemicals, flames or electric discharge	Prolonged time in direct contact with hot liquids, chemicals, flames or electric discharge
Appearance	Dry burns with erythema and desquamation Absence of blisters Blanch with pressure	Wet/weeping burns with erythema Blisters present Blanch with pressure	Moist burns with erythema and a red-waxy white appearance Blisters present easily unroof Delayed blanch when pressure is applied	Waxy white to dark-leathery dry and inelastic burns Do not blanch with pressure	White or black burns Do not blanch with pressure
Sensation	Painful	Extremely painful	Painful only with pressure	Painless unless deep pressure is applied	
Healing time	3–6 days	7–14 days	>21 days, usually require surgical treatment	Require surgical treatment to start healing	
Scarring	No scarring observed	No scarring, but skin dyspigmentation is expected	Hypertrophic and keloid scarring expected with or without skin contracture		Hypertrophic and keloid scarring with severe skin contracture

*SPTB* superficial partial-thickness burns, *DPTB* deep partial-thickness burn

## Review

### Search strategy

This review was carried out in two parts: (1) standard-of-care, second-degree burn dressings, and (2) pre-clinical, second-degree, hydrogel burn dressings. For the former, a search was carried out assessing standard-of-care second-degree burn dressings published between 1980–2021. Papers evaluating such burn treatments included both male and female, and adult and pediatric populations.

Inclusion and exclusion criteria for standard-of-care, second-degree burn dressings are outlined below.

Inclusion criteria

- Papers focusing only on the treatment of second-degree burns, including both SPTB and DPTB.
- Dressings included: traditional dressing pads (gauze, tulle gras dressings), silver sulfadiazine (SSD) and other antimicrobial-impregnated dressings (i.e. containing chlorhexidine), hydrocolloid dressings, silicon-coated dressings (i.e. biosynthetic silicon coated) and hydrofiber dressings. More dressings exist to manage second-degree burns; however, based on the literature, these dressings were the most frequently used to treat second-degree burns.

Exclusion criteria include

- Papers including treatments for first, third or fourth-degree burns.
- Case reports and case series.
- Non-human studies.

The latter part of this review, pre-clinical, second-degree, hydrogel burn dressings, includes a search that was implemented for hydrogel burn dressings from 2013–2021. The main search engines include PubMed, Web of Science and Elsevier libraries. The searches were conducted systematically to retrieve pre-clinical, second-degree, hydrogel burn dressings and their effects on (1) antimicrobial properties, (2) drug delivery modalities or (3) dissolvable systems.

The main outcomes of this search are bacterial zone of inhibition (ZOI) (antimicrobial properties), time of wound healing (drug delivery modalities), or pain and tissue damage (dissolvable systems).

Inclusion and exclusion criteria for hydrogel, second-degree burn dressings are outlined below.

Inclusion criteria

- Hydrogels for second-degree burn wounds.
- Properties including: antimicrobial, drug delivery, dissolvable.
- Outcomes: measured via bacterial ZOI, microbial inoculum, antibacterial activity (%), drug loading, cytokines, *in vivo* histopathology, burn wound healing rate, microvascular formation, dissolution time.

Exclusion criteria include

- First, third, or fourth-degree, hydrogel burn dressings.
- Studies involving antimicrobial, drug delivery, or dissolving hydrogels for a non-burn application.
- Studies that do not meet the inclusion criteria.

### Burn wound management

Burns, unlike other open wounds (e.g. abrasions or lacerations) differ in pathophysiology. When the skin suffers a burn injury, the heat effect causes increased capillary permeability with plasma leakage into the interstitial space (instead of causing edema and local inflammation). This accounts for the rapid loss of fluid and compromised availability of inflammatory mediators. Both the innate and adaptative immune response become limited. The burn wound becomes depleted of phagocytic cells, T cells and plasma cells responsible for mediating phagocytosis, intracellular killing, chemotaxis of other inflammatory cells and production of immunoglobulins for further immune protection [15]. Therefore, the risk of burn wound infection is high, especially secondary to ‘normal flora’ present in the epithelial appendages. Additionally, it is also important to recognize that burn wounds are susceptible to increased evaporative water loss due to destruction of the lipoprotein complex in the stratum corneum of the skin, responsible for acting as a barrier against evaporation. This evaporative loss can lead to dehydration, electrolyte abnormalities, hypothermia and increased metabolism [16]. Thus, caring for a burn wound begins with debridement followed by covering the wound to create an environment that promotes re-epithelization and prevents cellular dehydration and secondary infection.

Correctly classifying the depth of the burn alongside early clinical evaluation and management is the first step. Differentiation between SPTB, DPTB and third-degree burns may not be apparent in the first days after the injury. Moreover, these wounds can suffer from burn conversion, a poorly understood process, in which the thermal injury spreads from a superficial to a full-thickness burn [17,18].

For all burns, the goals of management and treatment include pain mitigation, prevention of infection and promotion of rapid healing, with the ultimate goal of restoring the injured region to both full function and visual aesthetic [7].

Second-degree burns, the most common burns resulting from thermal injuries, are often managed on an outpatient basis [19]. Multiple dressing options are currently available, and their use depends on the burn depth, volume of exudate present, cost, provider familiarity and patient comfort [20]. For the treatment of SPTBs and DPTBs, the gold standard is conventional low-cost gauze impregnated with SSD [7]. These dressings provide a temporary protective barrier until tissue integrity can be naturally restored. However, such dressings often adhere to the wound surface and delay the healing process due to frequent changes at the wound site and increased toxicity to the regenerating keratinocytes. Nonetheless, this concern does not appear to prevent their widespread clinical use.

As the understanding of dermal wound healing advances, the range of treatments available for second-degree burns has also expanded (Table 2) [6,7,14,20–22]. More recent dressings promote wound healing, absorb excess exudate, reduce bacterial burden and minimize pain during dressing changes [19]. In addition, some innovative dressings accommodate movement of the burned skin and facilitate the patient’s return to daily activities [19,23].

**Table 2:** Advantages and disadvantages of the most commonly used dressings to treat second-degree burns. Information from [6, 14, 21]

	**Advantages**	**Disadvantages**	**Examples of commercially available options^a^**
Dressing pads	• Low cost • Antibacterial protection • Ideal for clean and dry wounds	• Requires frequent dressing change and tape to secure the pad • Changing dressing disrupts the wound bed and may be painful • Not for wounds with high exudates	Xeroform™
Antimicrobial dressings	• Low cost • Minimize bacterial colonization	• Cytotoxicity may cause would healing delay • Constant removal may be traumatic, disrupting the granulation tissue	Acticoat™
Hydrocolloid dressings	• Semi-permeable molecules swell with exudates and form a gel to protect against bacteria and moisture • Can be easily detached • Ideal for areas of great friction	• Destruction of dressing results in unpleasant color and odor often confused with infection • Not capable of absorbing big amounts of exudate	Duoderm™, Urgotul™
Silicon-coated nylon dressings	• Easy and atraumatic removal • Protect new tissue growth	• Not for wounds with high exudates • Sensitivity has been reported to silicone	Mepitel™
Hydrofiber dressings	• Moist microenvironment promotes healing	• High cost • Destruction of dressing results in unpleasant color and odor often confused with infection	Aquacel Ag™
Hydrogels	• Outer surface impermeable to bacteria and water • Transparent structure allows wound visualization without dressing removal • Flexible and easy to detach • Assists in autolytic debridement	• Low absorption capacity usually demands secondary dressing • Maceration can occur if exudate is abundant	IntraSite™, Nu-Gel™

^a^Commonly used wound dressings for burns according to the American Academy of Family Physicians, The American Academy of Dermatology and Burn Care & Research [7, 20, 22]

The following section provides an insight into clinical trials evaluating different dressings used to address second-degree burns. Special attention is given to hydrogel dressings, as they have shown considerable advantages over traditional dressings [8].

#### Dressing pads

**Figure 4.**
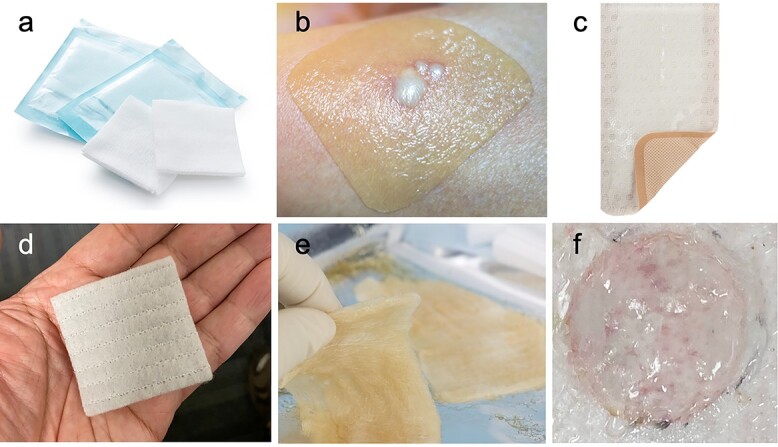
Representative images of wound closure materials. (a) Dressing pads, (b) hydrocolloid dressing, (c) silicon-coated nylon dressing, (d) hydrofiber dressing, (e) hydrogel dressing and (f) dissolvable hydrogel dressing. Images (a–e) were purchased from Shutterstock.com and image (f) via copyright permission obtained from Cook *et al*. [78]

Dressing pads (Figure 4a) refers to simple, medicated or nonmedicated, non-adherent dressings made of gauze (loosely woven translucent cotton fibers). Gauze pads, applied directly to the injured tissue, provide a protective barrier to infection while allowing oxygenation to promote healing. These dressings are sometimes combined with paraffin to avoid skin damage when removed (i.e. a tulle dressing). However dressing changes often result in pain and reinjury of the tissue, increased length of the re-epithelialization process and may require anesthetization of the patient [24,25].

As mentioned, dressing pads containing SSD are the current standard-of-care for second-degree burn injuries [7]. However, iodine and chlorhexidine are also used as antimicrobials, though less commonly. The major advantages of antimicrobial-containing gauze dressings are their low cost, widespread availability and effective prevention of local bacterial infection [7].

Local cytotoxicity of the silver ions to keratinocytes and fibroblasts along with pain eliciting from constant dressing changes are common following treatment of burns with SSD dressings [26–33]. Nonetheless, silver ions have been historically recognized as potent antimicrobials with cytotoxic activity against numerous bacteria, viruses, yeast and fungi, and therefore newer formulations with silver are being manufactured to maintain the broad antibacterial spectrum while causing less local toxicity [26,34]. Clinical trials comparing nanocrystalline silver (NC) to traditional SSD formulations for the treatment of deep partial-thickness burns show that NC dressings present lower incidence of infections in comparison to SSD formulations (9.5 *vs* 27.8% with an odds ratio of 0.14, 95% confidence interval [0.06–0.35]) [34]. Moreover, two studies report significantly less pain with Acticoat™ NC dressings compared to SSD dressings using a visual analog scale (VAS) for pain (1–10; 0 being no pain, 10 being severe pain) [35,36]. Varas *et al*. report a mean VAS pain difference of 3.2 for NC dressing *vs* 7.9 for SSD (*p* < 0,001), whereas Muangman *et al*. report a VAS difference of 4.0 ± 0.6 for NC dressing *vs* 5.0 ± 0.7 for SSD [35,36]. However, the authors of this meta-analysis conclude that despite the proven lower incidence of infections and higher satisfaction with NC dressing, further randomized studies are needed to confirm the results and change the current guidelines for the management of second-degree burns [35].

A weakness of antimicrobial-impregnated gauze pads is insufficient adsorption of wound exudate. Gauze dressings require daily changes that, as previously mentioned, often result in traumatized tissue as well as pain and discomfort to the patient. Chaganti *et al*. summarize three randomized controlled clinical trials in the USA and China comparing the rate of re-epithelization and time to wound healing of second-degree burns using traditional SSD with gauze *vs* highly absorptive foam dressings—dressings consisting of layers of semipermeable polyurethane manufactured specifically to absorb large amounts of exudate [19]. These absorptive foam dressings enhance autolytic debridement, provide a moist wound environment and promote healing [37–39]. According to Chaganti *et al*., healing periods for second-degree burns are similar regardless of the type of dressing used [19]. The data show no statistically significant difference in time to complete healing between using foam dressings and SSD with gauze (Table 3) [19]. In contrast to healing time, pain varies with the type of dressing. All trials used the Johns Hopkins VAS, but specific data collection timepoints varied between trials: Silverstein *et al.* report pain scores during dressing application, during wear and on dressing removal; Yang *et al*. report pain scores before wound treatment and after treatment at days 7, 14, 21 and 28; and Tang *et al*. report pain scores before, during and after dressing removal from week 1 to 4 [37–39]. Despite the differences in the clinical study designs, the use of foam dressings causes less pain and is more comfortable for patients, particularly at earlier stages of healing [19].

**Table 3:** Results from Chaganti *et al*.’s systematic review comparing traditional SSD with gauze and foam dressings for the treatment of second-degree thermal burns [19]

**Trial**	**Follow-up**	**Percentage of patients with complete healing (RR)**	**Time to complete healing**
Silverstein *et al.* [35]	21 days post burn or until full reepithelization	1.2 [95%CI, 0.95–1.53]	Mean days: 17 for SSD *vs* 13 for foam dressing (*p* > 0.05)
Tang *et al.* [33]	28 days post burn or until full reepithelization	1.0 [95%CI, 0.85–1.17]	Median days: 15 for SSD *vs* 16 for foam dressing (*p* 0.74)
Yang *et al.* [34]	28 days post burn	1.0 [95% CI, 0.87–1.2]	Mean days: 25 ± 4 SD for SSD *vs* 22 ± 3 SD (*p* < 0.05)

*SSD* silver sulfadiazine, *CI* confidence interval

#### Hydrocolloid dressings

Hydrocolloid dressings (Figure 4b) consist of a layer of gel-forming material adhered to a semi-permeable film or foam backing. This gel layer comprises an adhesive polymer matrix containing a combination of absorbent materials including gelatin, pectin and sodium carboxymethyl cellulose that absorb exudates and swell into a gel-like substance providing a moist environment. Hydrocolloids are waterproof, flexible dressings that assist in tissue regeneration and granulation, are impermeable to bacteria and promote autolytic debridement of dry and/or necrotic wounds [23]. Application and removal of hydrocolloid dressings is easier and less painful than most other dressing types. These dressings are available in a variety of sizes, shapes and thicknesses and may also include adhesive borders making them ideal for wounds in high-friction areas of the body, such as the sacrum, heels and elbows, wshere they reduce the likelihood of rucking, wrinkling or edge rolling [40]. In addition, some of these dressings may be transparent, allowing visualization of the wound without removal of the dressing. However, efficacy depends on the amount of exudate, as these dressings are not designed to treat wounds with high-volume exudates. Leakage and discharge of unpleasant color and odor, which is often mistaken for infection, can result if the wound presents with high exudate production, requiring more frequent dressing changes and overall compromising the cost-effectiveness of this product.

A systematic review comparing three randomized clinical trials reveals superior efficacy (time to complete healing) of hydrocolloid dressings over chlorhexidine-impregnated paraffin gauze dressings [23,41–43]. Moreover, the incidence of infection, adverse events and pain levels are also reduced in the hydrocolloid dressing group. Similarly, Wright *et al*., report higher satisfaction for the hydrocolloid dressings *vs* the chlorhexidine-impregnated paraffin gauze (satisfaction levels recorded for both investigators and participants using a 10-item VAS, with 0 = useless and 10 = excellent) [43]. Satisfaction levels rate higher for hydrocolloid dressings from both participants and investigators (Table 4) [43]. However, unlike what would be expected, the authors report a more frequent need to change the hydrocolloid dressings in comparison to gauze. The main reason is that patients treated with hydrocolloid dressings presented extensive leakage (15%) compared to those treated with the conventional gauze dressings (3%). The difference in reasons for dressing change between groups is significant (*p* = 0.01) and besides leaking, other reasons included pain, discomfort and detachment of the dressing. In addition, the authors report no significant difference in the difficulty between removing either of the dressings, questioning whether patient satisfaction could be diminished if they had to buy more dressings to treat the same wound.

**Table 4:** Satisfaction levels from Wright *et al.*’s study comparing hydrocolloids to clorhexidine-impregnated paraffin gauze [43]. Data reported using a 10 item visual analog scale

**Dressing**	**Participants**	**Investigators**
Hydrocolloid	9.04	9.31
Chlorhexidine-impregnated paraffin gauze	6.86	6.9
*P* value	*p* < 0.02	*p* = 0.05

#### Silicon-coated nylon dressings

Silicon-coated nylon dressings (Figure 4c) are flexible, porous, semitransparent polyamide nets coated with a silicone that facilitates the application and retention of the dressing in the wound area [23]. These highly pliable and stretchable dressings are amenable to placement on tissue with complex surface contours. The dressing’s open mesh structure protects the wound while allowing free passage of exudate into a secondary dressing, reducing frequent dressing changes. In addition, these dressings are non-adherent and therefore removal is facile and atraumatic [44].

It is important to note that some modern silicon-coated nylon dressings contain biologics, such as collagen peptides, and serve as biosynthetic skin substitutes [45,46]. The collagen component allows adhesion to fibrin on the clean wound surface and contributes to pain reduction, while the silicone outer layer allows some water loss to promote adequate moisture and induce healing [45].

Demling and DeSanti report the efficacy of topical antibiotic management with bacitracin *vs* TransCyte™, a commercially available biosynthetic silicon-coated dressing (on a nylon mesh coated with porcine collagen and newborn human fibroblast cells), for the treatment of mid-partial-thickness burns of the face [47]. The authors classified the study population as major burns (11 patients that required at least 7 days hospitalization) and minor burns (10 patients with criteria for outpatient care). The results indicate a significant decrease in the daily care time**,** pain between and during wound care (VAS, assessed pain from 0–10, with 0 being the lowest) and healing time (time to re-epithelization), favoring the skin substitute group (Table 5) [47]. However, despite satisfactory results, the authors conclude that the complex design associated with the use of live cells in this product in addition to the high cost of production decreases availability, and, therefore, the potential clinical translatability of this dressing [48].

**Table 5:** Comparison between topical agents and TransCyte™ skin substitute according to Demling and DeSanti’s study [47]

**Mean ± S.D.**	**Face care (h/day)**	**Pain scale**		**Healing time (days)**
		**During**	**Between**	
Major burns				
Topical agents	1.9 ± 0.5	5 ± 1	4 ± 2	14 ± 4
TransCyte™ skin substitute	0.35 ± 11^a^	2 ± 1^a^	2 ± 0.1^a^	8 ± 2^a^
Minor burns				
Topical agents	2.2 ± 0.4	5 ± 1	3 ± 2	12 ± 3
TransCyte™ skin substitute	0.4 ± 0.01^a^	2 ± 1^a^	1 ± 0.5^a^	8 ± 1

^a^Significantly diffferent from topical agent, *p* < 0.05

#### Hydrofiber dressings

Hydrofiber dressings (Figure 4d) are absorbent and biodegradable dressings prepared from sodium carboxymethyl cellulose specifically designed to treat moderate to heavily exudating wounds [49,50]. Similar to hydrocolloid dressings, hydrofiber dressings transform into a gel-like substance to create a moist microenvironment that promotes healing while limiting wound secretion and bacterial communication [51]. The advantages of these dressings include: (1) highly absorbent material, (2) mechanical stability and (3) ease of removal with saline irrigation, minimizing the pain and tissue damage during and after dressing changes. Muangman *et al*. describe the superior efficacy of hydrofiber dressings coated with ionic silver (Aquacel Ag™) for the treatment of partial-thickness burns in outpatients, as these dressings require less time for wound closure, reporting a difference in time of 3.7 days (95% confidence interval [1.9–5.4]) (Table 6) when compared to SSD dressings [52]. Similarly, patients report substantially fewer visits to the hospital for dressing change and less pain with hydrofiber dressings during dressing changes, showing a reduction of pain scores at days 1, 3 and 7 post-treatment (pain scores are registered on a 10 point VAS, with 0 representing no pain) (Table 6). Treatment with hydrofiber dressings is more cost-efficient than SSD dressings (including both hospital and travel costs) [52]. However, despite satisfactory results, this product remains expensive and may be unavailable due to low demand.

**Table 6:** Comparison between AquacelAg™ and SSD acoording to Muangman *et al*. [52]

**Dressing**	**AquacelAg ™**	**SSD**	***P* value**
Time to wound closure (days)	10 ± 3	13.7 ± 4.3	*p* < 0.02^*^
Number of hospital visits for dressing change	3.5 ± 1	13.7 ± 4	*p* < 0.001
Pain scores Day 1 Day 3 Day 7	4.1 ± 2.1 2.1 ± 1.8 0.9 ± 1.4	6.1 ± 2.3 5.2 ± 2.1 2.2 ± 1.9	*p* < 0.02
Total cost US$	52 ± 2	93 ± 36	*p* < 0.01

*SSD* silver sulfadiazine

#### Hydrogel dressings

Hydrogels (Figure 4e) are 3D networks of hydrophilic polymer chains that are water insoluble but swell in the presence of water [53]. These dressings are also transparent and can be fabricated into any shape to provide a moist environment for wound repair. Hydrogels are composed of either natural biopolymers such as alginate, collagen and chitosan or synthetic polymers such as polyvinyl alcohol or polyethylene glycol. Finally, hydrogels exhibit good permeability, easy degradation and excellent biocompatibility [54].

A prospective cohort study by Homann *et al*., compares the performance of a liposome polyvinyl-pyrrolidone-iodine (PVP-I) hydrogel (antimicrobial hydrogel) *vs* SSD cream in 43 patients with partial-thickness burn wounds [55]. The handling of the preparation and cosmetic outcomes (defined by smoothness, elasticity and appearance of wound) scale ranges from 1 (excellent) to 7 (very poor). Compared to SSD cream, the PVP-I hydrogel affords better handling and cosmetic results **(**Table 7). In addition, healing time also decreased with use of the liposome PVP-I hydrogel to 9.9 ± 4.5 days from 11.3 ± 4.9 in the SSD group (*p* < 0.015). [55] Similarly, Patel and Shah, compare a hydrogel dressing to SSD (applied over dry gauze) for the treatment of second-degree burns involving up to 25% of total body surface in 50 patients. The results support the use of the hydrogel as 56% of the patients in this group (*n* = 25) healed within 15 days, while 52% in the conventional group (SSD gauze, *n* = 25) healed within 21 days (*p* < 0.02) [56]. In addition, Patel and Shah note that 56% of patients had up to 25 applications of conventional dressing throughout the entire re-epithelization process, compared to 56% of patients that only required 5 re-applications of hydrogel dressing [56]. Overall, the hydrogel promotes faster re-epithelization and is easier to apply and remove while minimizing unnecessary pain or destruction of the already formed granulation tissue.

**Table 7:** Comparison between PVP-I hydrogel and SSD cream acoording to Homann *et al*. [55]

		**PVP-I Hydrogel**	**SSD cream**
Handling	Excellent	37%	13%
Cosmetic	Excellent	37%	13%

*PVP-I* Polyvinyl-pyrrolidone-iodine, *SSD* silver sulfadiazine

### Hydrogels in pre-clinical development for second--degree burn wounds: structures and dressing compositions

The following section emphasizes some of the most important features that make hydrogels ideal for the management of second-degree burns [8]. As mentioned above, hydrogels are hydrophilic, 3D, polymeric networks that swell upon exposure to an aqueous environment. Liang *et al*. describe an ideal wound dressing as one that is non-toxic, does not cause inflammation, retains moisture, absorbs wound exudate, maintains its physical and mechanical integrity and strength, avoids bacterial infection and promotes cellular functions such as cell adhesion, proliferation and differentiation [57]. Today, hydrogels under study perform relatively simple tasks (e.g. protecting the wound) to more complex ones, where cues are provided to direct a biological outcome, as described in the following sections. We begin with summarizing the crosslinking chemistry and the corresponding reactive groups required, followed by a discussion of the various functions (e.g. antimicrobial, drug delivery and dissolvable).

#### Hydrogel dressing structures and reactive groups

**Figure 5.**
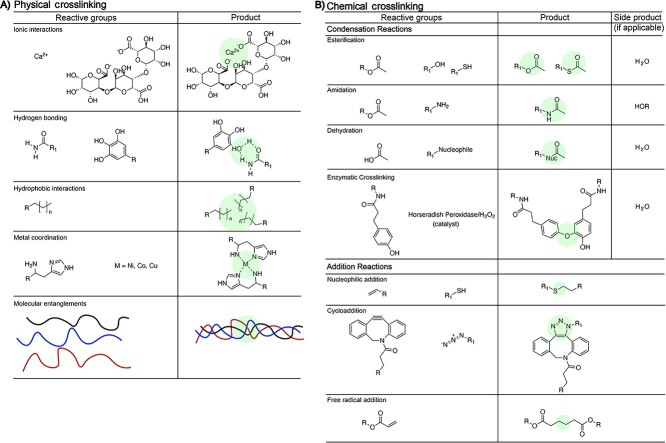
Hydrogel crosslinks, highlighted in green, representing examples of physical crosslinking (left) chemical crosslinking (right)

Hydrogels are comprised of natural and/or synthetic materials and are ideal candidates for the treatment of second-degree burns as they protect the burn from the outside environment, absorb excessive wound exudates and exhibit mechanical properties similar to skin. Hydrogels, as well as the materials that comprise them, are prepared through a variety of chemical and physical processes (Figure 5) and are primarily characterized via mechanical properties, weight percent, adhesion, swelling, gelation, gel fraction, morphology, degradation and cytotoxicity. As shown in Figure 5 (left), common physical crosslinking strategies involve hydrogen-bonding and metal or non-metal coordination between the polymer chains and physical entanglements of polymer chains. For example, alginate hydrogels are prepared via calcium crosslinking. Chemical crosslinking reactions also afford crosslinked hydrogels. Esterification, amidation and addition reactions are often used, especially with synthetics-based hydrogels. By changing the extent of physical or chemical crosslinking within the hydrogel, the physical and mechanical properties of the hydrogel change. For example, increasing the crosslinking density in a hydrogel will increase mechanical properties, reduce swelling and delay degradation. Furthermore, hydrogel burn wound dressings are prepared to target specific properties beneficial to supporting wound healing such as antimicrobial activity, drug delivery and/or degradation of the dressing.

#### Hydrogel dressings with antimicrobial properties

Antimicrobial-impregnated hydrogel dressings aim to minimize burn wound bacterial infection through three main modes: (1) utilizing antimicrobial chitosan polymers in the hydrogel backbone, (2) loading hydrogels with antibiotics or (3) locally delivering silver nanoparticles (AgNPs), among other antimicrobial agents, directly to the burn wound.

Chitosan-based hydrogels are widely used due to their antimicrobial activity. Chitosan hydrogels are prepared through physical crosslinking via hydrogen bonding or chemical crosslinking via modifying chitosan’s structure to include alkene or acrylic crosslinking functional groups. Chitosan’s innately antimicrobial activity arises from its ability to bind the negatively charged bacterial cell wall, causing disruption of the membrane and ultimately increasing permeability that culminates in the bacterial cell wall destruction [58]. Once the bacterial cell wall is compromised, chitosan complexes with bacterial DNA, inhibiting replication and resulting in apoptosis of the cell [58].

Specifically, Dang *et al*. report the bacterial ZOI, the area around the treatment where growth is inhibited, upon treatment with nanocurcumin-loaded (nCur), chitosan-pluronic (CP) copolymer hydrogels (nCur-CP). nCur-CP hydrogels show significant antimicrobial effects via an increased ZOI against *Escherichia coli*, *Salmonella typhimurium*, *Pseudomonas aeruginosa* and *Staphylococcus aureus* relative to curcumin treatment alone (Table 8) [59]. The thermosensitive copolymer is synthetically prepared via a substitution reaction [60]. Upon heating the polymer, linkages form between the N-H of the primary amine and the amide carbonyl groups of the chitosan, as observed via Fourier-transform infrared spectroscopy, to gel the polymer. This sol–gel transition to form the hydrogel occurs at temperatures >25°C, a temperature lower than that of human skin [60]. Additionally, the nCur-CP hydrogel copolymers exhibit similar antimicrobial effects to the commercially available antibiotic, chloramphenicol [59]. Similarly, gentamicin-loaded chitosan hydrogels (CS-GT) act as antimicrobial burn wound dressings against *S. aureus* and *P. aeruginosa* [61,62]. The CS-GT hydrogel promotes skin repair after a scalding burn and significantly increases the ZOI against *S. aureus* and *P. aeruginosa* (*p* < 0.05) compared to chitosan and gentamicin alone after 24 h of treatment at 37°C (Table 8) [61]. This study suggests that chitosan itself exhibits limited antimicrobial effects alone, the opposite finding of previous studies using chitosan as an antimicrobial [63–65]. Additionally, the sol–gel transition temperature of >25°C suggests that this hydrogel may not solidify in cold temperatures, limiting the application of this dressing to warm climates where the temperature does not decrease below 25°C.

**Table 8:** Zone of inhibition of antimicrobial hydrogels and their controls against *P. aeruginosa, S. aureus, E. coli and S. typhimurium* [59–61, 66]

**Hydrogel formulation**	***P. aeruginosa***	***S. aureus***	***E. coli***	***S. typhimurium***
nCur-CP	27 ± 1.2 mm	27 ± 0.5 mm	24 ± 0.3 mm	20 ± 0.5 mm
Curcumin	11 ± 0.5 mm	20 ± 1.0 mm	14 ± 0.3 mm	9 ± 0.2 mm
Chitosan	7.0 ± 1.0 mm	7.0 ± 1.0 mm	N/A	N/A
Gentamicin	21.3 ± 0.6 mm	17.7 ± 1.2 mm	N/A	N/A
CS-GT hydrogel	20.3 ± 1.0 mm	20.0 ± 1.0 mm	N/A	N/A
Chitosan-PVA hydrogel + AgNPs	N/A	10.2 ± 1.0 mm	9.7 ± 1.3 mm	N/A
Chitosan-PVA hydrogel	N/A	1.0 ± 0.5 mm	0.8 ± 0.6 mm	N/A
PVA-AgNP (4 h)	1.00 x 10^0^ CFU/ml	1.00 x 10^3^ CFU/ml	1.00 x 10^0^ CFU/ml	N/A
PVA-AgNP (48 h)	1.00 x 10^2^ CFU/ml	1.00 x 10^4^ CFU/ml	1.00 x 10^2^ CFU/ml	N/A
10% PVA gel (4 h)	3.73 x 10^7^ CFU/ml	3.73 x 10^7^ CFU/ml	3.73 x 10^7^ CFU/ml	N/A
10% PVA gel (48 h)	3.73 x 10^7^ CFU/ml	3.73 x 10^7^ CFU/ml	3.73 x 10^7^ CFU/ml	N/A
PVA powder (4 h)	3.73 x 10^7^ CFU/ml	3.73 x 10^7^ CFU/ml	3.73 x 10^7^ CFU/ml	N/A
PVA powder (48 h)	3.73 x 10^7^ CFU/ml	3.73 x 10^7^ CFU/ml	3.73 x 10^7^ CFU/ml	N/A

*P. aeruginosa* Pseudomonas aeruginosa, *S. aureus* Staphylococcus aureus, *E. coli* Escherichia coli, *S. typhimurium* Salmonella enterica serovar typhimurium

Reinhart and Campbell report a chemically crosslinked chitosan- polyvinylalcohol (PVA) hydrogel (15:85 wt% chitosan:PVA) loaded with 5 mM antimicrobial AgNPs prepared with glutaraldehyde as the crosslinking agent. The AgNP-loaded chitosan-PVA hydrogel significantly decreases bacterial growth of *S. aureus* and *E. coli* as compared to a chitosan-PVA dressing without AgNPs based on ZOI (Table 8) [66]. The low ZOI of the chitosan-PVA hydrogel alone suggests that chitosan and PVA have minimal antimicrobial activity against *S. aureus* and *E. coli*. Jackson *et al.* suggest that chitosan is not necessary for the antimicrobial effects and instead the AgNPs are necessary to maintain antimicrobial properties. Jackson *et al*. utilize PVA-based hydrogels chemically crosslinked with silver nitrate via a substitution reaction forming a PVA-based hydrogel loaded with AgNPs (PVA-AgNP) [67]. The resulting PVA-AgNP hydrogel significantly inhibits bacterial growth compared to both PVA hydrogels and PVA powder alone (Table 8) [67]. However, after 48 h exposure of the PVA-AgNP dressing to bacteria, there is an increase in the number of bacteria present (Table 8). This suggests that this burn wound dressing is not a long-term, sustainable solution in preventing bacterial infection in burn wounds as the dressing would require changing within 48 h of initial application. Dressing changes cause damage to the newly forming tissue on the burn wound due to the adhesion of the dressing to the tissue, thus limiting the applicability.

Likewise, Boonkaew *et al*. report an AgNP-loaded hydrogel prepared via an addition reaction using ultraviolet (UV)-irradiation from 2-acrylamido-2-methylpropane sulfonic acid sodium salt (AMPS) and *N-N′*-methylenebisacrylamide (MBA) (AMPS-MBA) [68]. After a 24 h treatment, the AgNP-loaded AMPS-MBA hydrogels prevent growth of *S. aureus*, MRSA and *P. aeruginosa* (>6.01 ± 0.00, 6.28 ± 0.00 and 7.26 ± 0.00 (log reduction), respectively) relative to commercially available Acticoat™. The AgNP-loaded AMPS-MBA hydrogels also show bacterial growth inhibition at 3 h, whereas Acticoat inhibits bacterial growth within 30 min.

Kim *et al*. describe a thermosensitive, injectable methylcellulose (MC) hydrogel containing AgNPs (MC/AgNP) as an antimicrobial burn wound dressing [69]. The MC/AgNP hydrogels are prepared through sol–gel transition, induced by hydrophobic interactions via a temperature increase. MC/AgNP hydrogels at both 0.5 and 1.0 wt% AgNP concentration exhibit 99.9% antibacterial activity against *Klebsiella pneumoniae*, *E. coli* and *S. aureus,* while MC hydrogels without AgNPs demonstrate no bacterial growth inhibition under the same experimental conditions. The antimicrobial properties of the MC/AgNP hydrogels are due to Ag^+^ from the silver nanoparticles binding to and penetrating bacteria cell walls, disrupting their structural integrity, increasing permeability and ultimately resulting in bacteria cell destruction [69]. This hydrogel shows bacterial inhibition, however there remains a concern regarding stability of the hydrogel due to its thermosensitive properties, such that the hydrogel may only be applicable in a particular climate zone(s).

Huang *et al*. describe an antibacterial cryogel, a hydrogel prepared at low temperatures, composed of gelatin (GT) and AgNPs [70]. An amidation reaction between *N*-hydroxysuccinimide (NHS)-ester and amine groups on the GT structures crosslinks the GT at −12°C over 36 h. Soaking the prepared GT cryogels in an AgNP solution for 2 h followed by lyophilization provides the AgNP-loaded GT cryogel (GT + AgNP). The GT + AgNP samples exposed to MRSA and *P. aeruginosa* prevent bacterial growth over a 24 h period, while ~60% growth occurs in the *E. coli* samples relative to the *E. coli* alone control group [70]. In addition to antimicrobial properties, these cryogels show a stress of <1 kPa at 20% strain, indicating a very weak gel. The GT cryogel is biodegradable and enzymatically degrades over 4 weeks *in vivo*. Since the hydrogel is weak, non-adherent and degrades over time, its removal from the wound minimizes new tissue damage. These hydrogels are also fabricated prior to application on the burn wound, and thus, require a second bandage to hold them in place.

Light-activated hydrogels, as reviewed by Maleki *et al*., produce reactive oxygen species to exert an antibacterial effect. ZnO nanoparticles, black phosphorus-, sinoporphyrin sodium- and TiO_2_-based hydrogels produced reactive oxygen species upon light irradiation [71]. For example, black phosphorus-loaded hydrogels provide antibacterial activity of up to 99.5% against *S. aureus* and 98.9% against *E. coli* [71]. Of the antibacterial agent-loaded hydrogels, the TiO_2_-based hydrogels exhibit the least antibacterial activity upon exposure to the visible light range, however potent antibacterial activity is observed under UV light. However use of UV light is a disadvantage as it is known to cause skin damage.

Hydrogels with antibacterial activity exhibit promise in preventing bacterial infection in burn wounds. However, adhesive properties, the strength of hydrogels and absorption of wound exudates are characteristics requiring further integration into these dressings to achieve an ideal burn wound dressing.

#### Hydrogel dressings—drug delivery systems

Drug delivery by way of hydrogels remains a challenge due to the large pore size of hydrogels (μm) resulting in fast initial drug release, described as a burst release. This burst release is an advantage and or a disadvantage depending on the intended application. Johnson *et al*. report an ibuprofen-encapsulated hydrogel as a burn wound dressing, prepared via pressurized gas to expand liquid-processed alginate hydrogel scaffolds (PGX technique). The PGX hydrogel is formed through ionic interactions between alginate and the calcium dication. The PGX preparation loads up to 8 wt% ibuprofen as compared to 0.0 ± 0.7 wt% in conventionally synthesized hydrogels [72]. This ibuprofen-loaded alginate hydrogel reduces discoloration and scabbing/hardness at day 3 and accelerates overall burn wound healing as early as day 14, while untreated burn wounds and those treated with alginate hydrogels alone do not heal until day 28. The reduction in the wound healing time is attributed to ibuprofen’s anti-inflammatory properties [72]. Additionally, it is proposed that healthy granulation tissue growth is promoted by an ion exchange between calcium and sodium ions in the alginate dressing and the wound, respectively, ultimately stimulating mitosis [72]. Hydrogels prepared through ionic interactions are generally weaker than chemically crosslinked hydrogels, with lifetimes between days to months depending on the extent of ionic crosslinking. While the ibuprofen-loaded alginate hydrogels reduce discoloration and scabbing/hardness, these hydrogels are prepared prior to application on the wound and require an additional dressing such as tape, gauze or bandage for adherence to the burn wound.

Zheng *et al*. describe a histatin-1 (His-1)-loaded gelatin hydrogel photo-crosslinked *in situ* with acrylic acid-modified cyclodextrin, allowing for the addition reaction of the alkenes on the acrylic moieties. The hydrogel is loaded with resveratrol (Res) to promote vascularization, reduce inflammation and act as an antioxidant to eliminate reactive oxygen species in burn wounds [73]. *In vitro,* the Res/His-1/gel increases angiogenesis and primary human umbilical vein endothelial cell migration after incubation for 5 and 10 h, respectively. *In vivo,* second-degree burn wounds treated with the Res/His-1/gel exhibit replacement of granulation tissue with healthy epidermis by day 7, as compared to no epidermis present at day 7 in the control groups [73]. Additionally, skin appendages and sebaceous glands develop in the Res/His-1/gel group by day 14, but do not form in control groups (Table 9) [73]. The Res/His-1/gel exhibits promising anti-inflammatory properties, as well as promoting angiogenesis both *in vitro* and *in vivo*. The acrylic moieties used to crosslink these hydrogels require an initiator and light source to expedite gelation. Based on the initiator, the light source will be UV or visible. Visible light is advantageous as it less harmful to the tissue.

**Table 9:** Drug-loaded hydrogel formulations and their time to healing defined as the time at which healthy appendages develop [73]

**Hydrogel formulation**	**Drug**	**Days to heal**
Gelatin + acrylic acid-modified cyclodextrin	Resveratrol and histatin-1	14
Alginate	Ibuprofen	14
Hyaluronic acid, dextran and β-cyclodextran	Resveratrol and pDNA encoding vascular endothelial growth factor	14
Chitosan and PVA	Silver nanoparticles and sildenafil citrate	14

*PVA* Polyvinylalcohol

Similarly, Wang *et al*. deliver biocompatible Res and plasmid DNA (pDNA) encoding vascular endothelial growth factor via hydrogels to reduce inflammation and promote angiogenesis [74]. The hydrogel is composed of methacrylate-modified hyaluronic acid, methacrylated dextran (Dex) and methacrylated β-cyclodextran (β-CD) which photochemically crosslink upon UV-irradiation. Polyethyleneimine-conjugated pDNA and Res is encapsulated into the hydrogel scaffold and accelerates burn wound healing in an *in vivo* rat model via inhibition of inflammation and by promoting microvascular formation. Healing rates of burn wounds treated with the hydrogel alone, a Res-loaded hydrogel and the Res/pDNA-loaded hydrogel all significantly increase by days 14 and 21, with the fastest wound closure time observed in the Res/pDNA-loaded hydrogel treatment group compared to the no treatment group [74]. The best performing hydrogel, the Res/pDNA-loaded hydrogel, shows promising burn wound healing properties, including inhibition of the inflammatory cascade as well as angiogenesis. However, utilizing UV radiation is a concern with regards to further tissue damage if crosslinking is performed *in situ*. A potential improvement in the system would be the targeted delivery of the pDNA to a specific cell type.

Samadi *et al*. utilize a chitosan/PVA-crosslinked hydrogel film loaded with AgNPs, for antimicrobial treatment, and sildenafil citrate (SC) for its pro-angiogenesis properties [75]. Hydrogels are loaded with SC via swelling in a 5% aqueous SC solution, and burns are treated immediately after swelling. They treated *in vivo* burn wounds with SC/AgNP-hydrogels, AgNP-hydrogels and SC-hydrogels, and assessed healing by time and appearance. The SC/AgNP-hydrogel group exhibits complete skin epithelial remodeling by day 14, while scabs remain on wounds in both the AgNP-hydrogel and SC-hydrogel treatment groups at day 14. Additionally, the SC/AgNP-hydrogel group shows fewer inflammatory cells than the other treatment groups. Collagen type III levels increase, as determined by silver staining, at day 4 in burns treated with SC/AgNP-hydrogels as compared to minimal collagen type III detected in the AgNP-hydrogel and SC-hydrogel groups. The presence of type III collagen is an indicator for a scarless wound. Additionally, the SC-AgNP-hydrogel treatment group increases angiogenesis as compared to the AgNP-hydrogel and SC-hydrogel groups. Overall, the SC/AgNP-hydrogel treatment group outperforms the controls with regards to *in vivo* accelerated epithelialization and tissue regeneration. Gelation of hydrogels prior to application on burn wound dressings minimizes adhesion of the hydrogel to tissue, and, thus a secondary bandage is needed to secure the hydrogel to the burn wound.

Although these drug-loaded hydrogels show promise as novel burn wound dressings, rigorous preclinical validation of efficacy needs to be conducted to determine the optimal formulations that will ensure and promote wound healing, adhere to tissue and limit UV photo-crosslinking in the clinical setting.

#### Dissolvable hydrogel dressings

Dissolvable hydrogels are advantageous burn wound dressings because they minimize the mechanical debridement that occurs during a dressing change and therefore protect the newly formed granulation tissue. These hydrogels disassemble upon exposure to an external stimulus. Konieczynska *et al*. and Cook *et al*., describe a polyethyleneglycol (PEG)-based dissolvable hydrogel [76–78]. These thiol–ester hydrogels are prepared through a carbonyl substitution reaction of a disubstituted PEG NHS ester and a branched polymer containing terminal thiols. Alternatively, the hydrogels are prepared through a carbonyl substitution reaction of a disubstituted PEG NHS ester and a branched polymer containing terminal amines, in which the PEG contains an internal thioester. The hydrogels subsequently dissolve via thiol–thioester exchange between the terminal thiol on a cysteine methyl ester (CME) and the thioester linkage within the hydrogel network [76,78]. Thiol–thioester exchange reaction with CME occurs at a physiologically relevant pH (7.4) and exhibits increased reaction kinetics at basic pH. The CME concentration controls the dissolution time, where increasing the CME concentration from 0.3 to 0.5 M at pH 7.4 decreases the dissolution time from 1 h to 36 min, respectively [79]. Cook *et al.* demonstrate that increasing the pH of 0.3 M CME to 8.6 further decreases the dissolution time of dissolvable hydrogels via thiol–thioester exchange to <10 min [78]. Dissolution time also increases as a function of hydrophobicity by lengthening the methylene chain lengths of the crosslinker structure from 1 to 5 to 10 methylenes within the PEG. Dissolution occurs in ~10 , 20 and 80 min for hydrogels containing methylene chain lengths of 1, 5 and 10, respectively (Table 10) [78]. Hydrogels with the same structure, but lacking a thioester functional group, do not exhibit dissolution, confirming the role of thiol–thioester exchange in the dissolution mechanism. Finally, results from an *in vivo* porcine second-degree burn model show functional performance with healing equivalent to conventional treatments with the added benefit of facile dressing change via dissolution.

**Table 10:** Hydrogel dissolution times based on pH and weight percent [76–80]

**Dissolvable linkage**	**Methylene chain length**	**pH**	**Dissolution solution**	**Dissolution time**
Thioester (15 wt%)	1	7.4	0.3 M CME	1 h
Thioester (15 wt%)	1	7.4	0.5 M CME	36 min
Thioester (15 wt%)	1	8.6	0.3 M CME	10 min
Thioester (15 wt%)	5	8.6	0.3 M CME	20 min
Thioester (15 wt%)	10	8.6	0.3 M CME	80 min
Ester (10 wt%)	2	7.4	PBS	4 h
Ester (15 wt%)	2	7.4	PBS	8 h
Ester (20 wt%	2	7.4	PBS	24 h
Diselenide bridge (30 wt%)	N/A	N/A	3 wt% H_2_O_2_	15 min
Diselenide bridge (30 wt%)	N/A	N/A	DTT (did not disclose weight percent)	Did not disclose dissolution time

*CME* cysteine methyl ester, *DTT* dithiothreitol

Cook *et al*. also report a dissolvable hydrogel containing internal ester linkages, susceptible to degradation via hydrolysis [79]. The hydrogel contains a PEG backbone in the crosslinker, reacted with succinic anhydride to provide internal esters, capped with NHS functional groups. This crosslinker is reacted with amine functional groups on either a 4-arm PEG-NH_2_ or poly(ethylenimine) (PEI) macromere, resulting in a carbonyl substitution reaction allowing a hydrogel to form. Upon hydrogel formation at 10, 15 or 20 wt%, degradation occurs over time as the hydrogel swells in water. The local pH of the hydrogel, prepared from PEI, catalyzes hydrolysis and the dressing degrades within 4–24 h as compared to hydrogels prepared with a 4-arm PEG-NH_2_ macromer that degrades in 7 days. This hydrogel formulation is a promising burn wound dressing as it eliminates the need for mechanical debridement during dressing changes.

Similarly, Lu *et al*. describe a dissolvable hydrogel as a burn wound dressing containing a PEI backbone with selenide reactive end groups [80]. Crosslinking in this system occurs through a condensation reaction by formation of intra- and inter-diselenide bridges upon oxidation on exposure to air, thus releasing H_2_. Diselenide hydrogel dissolution occurs via two mechanisms, (1) oxidation of the diselenide bonds upon exposure to excess 3 wt% H_2_O_2_ solution for 15 min or (2) reduction of diselenide bonds using dithiothreitol (Table 10). Diselenide hydrogels applied to *ex vivo* porcine tissue and subsequently exposed to H_2_O_2_-soaked gauze dissolve after 30 min. Diselenide hydrogels are a promising burn wound dressing as the dissolution capability will minimize the need for the painful, mechanical debridement that damages new tissue growth.

Huang *et al*. report a hydrogel burn wound dressing prepared from carboxymethyl chitosan (CMC) and oxidized cellulose nanocrystal through a reversible Schiff-base bond between the chitosan and the aldehyde on the oxidized cellulose nanocrystal [81]. This hydrogel dressing dissolves on demand using an amino acid solution with primary amines that compete with chitosan, allowing another Schiff-base linkage to form and therefore disrupting the hydrogel 3D structure. Dissolution of this hydrogel allows for ease of dressing removal, minimizes pain with dressing changes and therefore diminishes the capacity to damage to newly formed tissue.

On-demand dissolution of hydrogels is a vital characteristic for burn wound dressings to avoid new tissue damage during dressing removal. The above dissolvable hydrogels demonstrate a new concept and show promise for further development and clinical evaluation.

## Conclusions

This review provides an overview of current practices and novel developments in the field of second-degree burn wound dressings. Specifically, it summarizes methods to treat second-degree burn wounds using polymeric hydrogels as dressings. From a design perspective, burn wound dressings should adhere to tissue, protect newly formed granulation tissue, possess antimicrobial activity, be easily removed and replaced and promote wound healing. Current pre-clinical hydrogels for burn wound dressings: (1) exhibit antimicrobial properties through use of SSD, antimicrobial polymers such as chitosan and NaNPs; (2) deliver ibuprofen to reduce inflammation, Res and His-1 to promote antiinflammation and pro-angiogenic properties, plasmid VEG-encoded DNA to promote angiogenesis and reduce inflammation, and sildenafil citrate for increased angiogenesis; and (3) dissolve upon demand to facilitate easier dressing changes in order to minimize mechanical debridement, pain and anesthesia. Significant research and development opportunities still exist with regards to: (1) optimization of the delivery of encapsulated anti-inflammatory, pro-angiogenic and antibiotic agents or the combined delivery of two or more agents; (2) encapsulation of cells or biologics; (3) methods of dressing removal which are pain-free, quick and facile; (4) incorporation of diagnostics to monitor wound healing; and (5) use of on-site 3D printers to fabricate dressings specific to patient requirements at the clinic or hospital (i.e. personalized dressings). Further, current practice is shifting the care of most burns to the outpatient setting where pharmacologic pain interventions and dressing-change frequency come up against practical barriers. This practice change creates an additional need and opportunity. Hydrogel burn dressings are a unique tailored solution for the management of burns and hold significant promise for improving patient care.

## Abbreviations

AgNPs: silver nanoparticles; AMPS: 2-Acrylamido-2- methylpropane sulfonic acid sodium salt; β-CD: β-Cyclodextran; CMC: Carboxymethyl chitosan; CME: Cysteine methyl ester; CP: Chitosan-pluronic; Dex: dextran; DPTB: Second-degree deep partial-thickness burn; GT: Gelatin; His-1: Histatin-1; MBA: *N-N′*-Methylenebisacrylamide; MC: Methylcellulose; NC: Nanocrystalline silver; nCur: Nanocurcumin; NHS: *N*-Hydroxysuccinimide; pDNA: Plasmid DNA; PEG: Polyethyleneglycol; PGX: Pressurized gas to expand; PVA: Polyvinylalcohol; PVP-I: Polyvinyl-pyrrolidone-iodine; Res: Resveratrol; SPTB: Second-degree superficial partial-thickness burn; SSD: Silver sulfadiazine; UV: Ultraviolet; VAS: Visual analog scale; ZOI: Zone of inhibition.

## Funding

We thank the NIH (R01EB021308) and Boston University for funding and supporting this research.

## Authors’ contributions

KAC and EM-L wrote the manuscript with comments and edits by EKR, AN, RS and MWG.

## Competing interests

KAC, AHC and MWG are listed as co-inventors on patents describing a technology for a dissolvable hydrogel dressing which is owned by the university. The patent is available for licensing.
